# Expression of CYLD and NF-*κ*B in Human Cholesteatoma Epithelium

**DOI:** 10.1155/2010/796315

**Published:** 2010-04-21

**Authors:** Jae Yong Byun, Tae Young Yune, Jee Youn Lee, Seung Geun Yeo, Moon Suh Park

**Affiliations:** ^1^Department of Otolaryngology, School of Medicine, Kyung Hee University Hoegi-dong, Dongdaemun-gu, Seoul 130-701, South Korea; ^2^Age-Related and Brain Diseases Research Center, Kyung Hee University, Seoul 130-701, South Korea

## Abstract

The tumor suppressor CYLD is a deubiquitinating enzyme that inhibits activation of the NF-*κ*B, which has key roles in inflammation and apoptosis. We hypothesized that CYLD may regulate the NF-*κ*B signaling pathway in cholesteatoma. We conducted immunohistochemistry to examine the expression of CYLD and NF-*κ*B in 16 cases of cholesteatoma and paired cases of retroauricular (RA) skin. In cholesteatoma epithelium, activated NF-*κ* B expression was significantly higher than in RA skin, whereas CYLD expression was significantly lower in cholesteatoma epithelium than in RA skin (*P* < .05). Furthermore, a significant inverse correlation was detected between CYLD and activated NF-*κ*B expression in cholesteatoma epithelium (*r* = −0.630). We found that CYLD reduced and activated increased NF-*κ*B in cholesteatoma epithelium in comparison to RA skin. The inverse correlation between CYLD and activated NF-*κ*B in cholesteatoma may be involved in cholesteatoma epithelial hyperplasia.

## 1. Introduction

The growth of keratinocytes in the epidermis is regulated by a delicate balance between molecules that control cell survival and cell death. If this regulation is disturbed, epithelial cells may become pathological hyperproliferative lesions, such as cholesteatoma. The molecular and cellular processes resulting in the clinical hallmarks of cholesteatomas (i.e., migration, uncoordinated proliferation, altered differentiation, and aggressiveness) are not yet fully understood. In cholesteatoma, keratinocytic differentiation and proliferation may be modulated by many cytokines, transcription factors, and inflammatory mediators such as TNF-*α* and IL-1*α* *[* *1* *]*. The tumor suppressor gene CYLD, which was recently identified as the cylindromatosis gene, has a deubiquitinating enzyme (DUB) activity and inhibits activation of the transcription factor NF-*κ*B, which has key roles in inflammation, immune responses, carcinogenesis, and protection against apoptosis [1]. In cholesteatoma, however, the role of NF-*κ*B has yet to be clearly defined. For example, NF-*κ*B was reported to be found in cholesteatoma epithelium, but it appeared to be inactivated [2]. However, a recent report suggested that activated NF-*κ*B plays a role in cellular hyperplasia of cholesteatoma [3]. Generally, in normal resting cells, NF-*κ*B is localized within the cytoplasm as a heterodimeric complex composed of p50 and p65 subunits bound to members of the class of inhibitory proteins called inhibitor kappa B (I*κ*B). After various stimuli, the phosphorylation of I*κ*Bs, an important step in NF-*κ*B activation, is mediated by I*κ*B kinase (IKK). The IKK complex consists of at least three subunits, including the kinases IKK-*α* and IKK-*β* and the regulatory subunit IKK-*γ*. Phosphorylated I*κ*B is then ubiquitinated, which targets it for degradation by the 26S proteasome, thereby releasing NF-*κ*B dimers from the cytoplasmic NF-*κ*B–I*κ*B complex and allowing them to translocate to the nucleus and induce target gene expression [4, 5]. At this point, CYLD binds to NF-*κ*B essential modulator (NEMO)/IKK-*γ* and appears to regulate its activity through deubiquitination of TNF receptor-associated factor 2 (TRAF 2) [5]. CYLD was recently reported to negatively regulate NF-*κ*B signaling by deubiquitination, and loss of deubiquitinating activity of CYLD is correlated with tumorigenesis [5–7]. CYLD also negatively regulates the c-Jun N-terminal kinase (JNK) signaling pathway and mitogen-activated protein kinase (MAPK) pathway, which are known to participate in a wide range of cellular processes, including proliferation, differentiation, and apoptosis of cholesteatoma [1, 8]. In addition, CYLD negatively regulates RANK signaling, which is known to be a key factor in bone resorption in cholesteatoma [9]. Therefore, investigation of CYLD expression may be a key step to understanding the cellular survival or hyperplasia in cholesteatoma epithelium. We hypothesized that CYLD may be involved in the NF-*κ*B signaling pathway and that the inverse correlation between CYLD and activated NF-*κ*B level may be related to the mechanism of cellular hyperplasia in cholesteatoma epithelium. To determine the role of CYLD in NF-*κ*B signaling of cholesteatoma, CYLD and NF-*κ*B expression levels in middle ear cholesteatoma epithelium were examined by immunohistochemical analysis to determine protein level and localization and compared to those of normal retroauricular (RA) skin. We also examined the relationship between CYLD expression and NF-*κ*B activation in middle ear cholesteatoma.

## 2. Methods

### 2.1. Subject Selection

Tissues of acquired cholesteatoma, diagnosed clinically and confirmed pathologically after surgery, were used in this study. All cholesteatomas extended to the mastoid, with varying degrees of attic bone destruction with keratin retention. The samples were obtained from patients ranging in age from 45 to 60 years (mean age: 52 years) who had undergone middle ear surgery for cholesteatomatous otitis media between November 2008 and May 2009 at the Department of Otolaryngology, Kyung Hee University East-West Neo Medical Center, Seoul, Korea. Paired normal RA skin was obtained during ear surgery for use as a control. Sixteen samples of cholesteatoma and paired normal RA skin samples were obtained for this study. The institutional review boards (IFC/IRB) of the Kyung Hee University East-West Neo Medical Center approved the protocol used in this study (IRB approval no. KHNMC IRB2008-007).

### 2.2. Immunohistochemistry

Cholesteatoma and normal RA skin were fixed in 4% formaldehyde, dehydrated in a graded ethanol series (70%, 95%, and 100%), and finally embedded in paraffin. Paraffin-embedded tissues were cut into 10 *μ*m thick sections using a paraffin block cutter (Thermo Shandon, Runcorn, Cheshire, UK) and mounted on adhesive slides coated with poly-l-lysine (cat. no. P8920; Sigma, St. Louis, MO). For immunohistochemistry, sections were deparaffinized, rehydrated, and treated in 10 mmol/L citric acid buffer (pH 6.0) at 95°C–100°C for 20 minutes for antigen retrieval. Endogenous peroxidase activity was blocked by incubation in 0.5% H_2_O_2_ in methanol for 20 minutes. Sections were then processed for immunohistochemistry using antibodies against CYLD (1 : 500 dilution; Abcam Inc., Cambridge, MA) and NF-*κ*B (1 : 300 dilution; Santa Cruz Biotechnology, Santa Cruz, CA) overnight at 4°C. The ABC method was used to detect cells labeled specifically using a Vectastain kit (Vector Laboratories, Burlingame, CA). Diaminobenzidine (DAB) was used as a substrate for peroxidase. The sections were subjected to a single immunostaining by enzyme-antibody method as described above. To confirm the colocalization of CYLD and NF-*κ*B, two serial sections (10–20 *μ*m thickness) were subjected to single immunostaining using each antibodie. Images were obtained using an Olympus (Tokyo, Japan) microscope and SPOT (Diagnostic Instruments Inc., Sterling Heights, MI). In all immunohistochemical controls, reaction to the substrate was absent if the primary antibody was omitted or if the primary antibody was replaced by nonimmune control antibody. Some sections were counterstained with hematoxylin to visualize the epithelial cell nuclei.

### 2.3. Data Analysis

Three investigators blinded to the experimental groups examined immunostained sections with an image analyzer attached to a light microscope. The total number of cells in the fixed domain, the number of cells with nuclear staining for NF-*κ*B and CYLD-positive cells in the epithelial layer of cholesteatoma and normal RA skin tissues, was counted under five ×400 fields in the fixed domain. Then, both the levels of activated NF-*κ*B (number of cells with nuclear staining/total number of cells counted) and CYLD expression (number of positively stained cells/total number of cells counted) were obtained and expressed as percentages.

 Data are expressed as means ± SD. The Mann-Whitney test was used to compare the means of paired variables. The level of significance was set at *P* < .05. The Pearson's correlation test was used to determine the association between activated NF-*κ*B and CYLD, with the level of significance set at 0.05. We also analyzed activated NF-*κ*B and CYLD levels according to the existence of active otorrhea and state of bony destruction using the Mann-Whitney test. The SPSS10 software package (SPSS, Chicago, IL) was used for the analyses.

## 3. Results

### 3.1. Immunohistochemistry of CYLD and NF-*κ*B Expression in Cholesteatoma Epithelium

**Figure 1:**
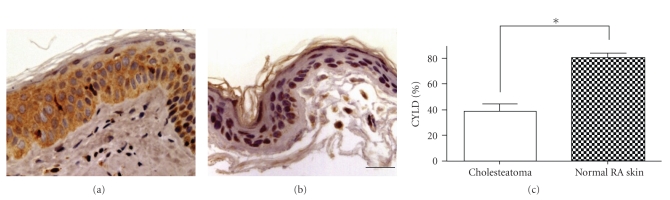
Immunohistochemistry of CYLD in cholesteatoma epithelium and normal retroauricular (RA) skin. (a): CYLD in normal RA skin. (b): CYLD in cholesteatoma epithelium. Note that strong CYLD immunoreactivity was observed mainly in the cytoplasm of cells in the basal and suprabasal epithelial layers of normal RA skin, whereas weak expression was detected in the cytoplasm of cells in the basal layer of the cholesteatoma epithelium. Bar = 300 *μ*m. (c): Rates of positive labeling for CYLD in cholesteatoma epithelium and normal RA skin. The rate of cells positive for CYLD was significantly lower in cholesteatoma epithelium than in normal RA skin. Data are presented as means ± SD. *P < .05.

The following results were obtained by immunohistochemical analysis of 16 cholesteatoma tissues and paired normal RA skin specimens. Immunohistochemical data indicated that immunoreactivity of CYLD was mainly present in the cytoplasm and perinuclear region of the basal layer and granular layer in normal RA skin (Figure 1(a)). In the cholesteatoma tissue, CYLD expression was seen in a few cells in the basal layer (Figure 1(b)). The percentage of CYLD-positive cells in cholesteatoma tissues was 38.48% ± 21.20% compared to 80.95% ± 8.08% in paired normal RA skin. The percentage of CYLD-positive cells in cholesteatoma tissue was significantly lower as compared to paired normal RA skin (*P* = .022).

**Figure 2:**
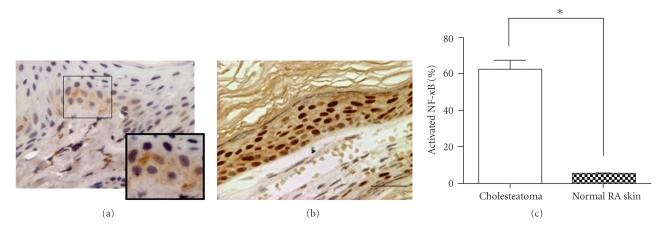
Immunohistochemistry of NF-*κ*B in cholesteatoma epithelium and normal retroauricular (RA) skin. (a): NF-*κ*B in normal RA skin. (b): NF-*κ*B in cholesteatoma epithelium. Note that strong expression was observed in the nuclei of cells in the basal and suprabasal layers of cholesteatoma epithelium, whereas weak NF-*κ*B immunoreactivity was mainly seen in the cytoplasm of cells in the basal epithelial layers of normal RA skin. Bar = 300 *μ*m. (c) Rates of positive labeling for NF-*κ*B in the nuclei of cells in cholesteatoma epithelium and normal RA skin. The rate of cells positive for NF-*κ*B was significantly higher in cholesteatoma epithelium than in normal RA skin. Data are presented as means ± SD. *P < .05.

 The differential expression of NF-*κ*B was found between normal RA skin and cholesteatoma epithelium. As shown in Figure 2(a), NF-*κ*B expression was seen mostly in the cytoplasm (inactivated form) and was localized mainly in the basal epithelial layer of normal skin. In addition, we found some nuclear staining of NF-*κ*B in the basal and suprabasal layers of the normal RA skin in some cases. In cholesteatoma tissue, however, most NF-*κ*B expression was observed in the nucleus (activated form) and was localized in all layers of the epithelium (Figure 2(b)). The percentage of activated NF-*κ*B expression in the cholesteatoma epithelium was 62.62% ± 18.97% and that in paired normal RA skin was 5.47% ± 1.12%. The percentage of activated NF-*κ*B expression in the cholesteatoma epithelium was significantly higher compared to normal RA skin (*P* = .0001). 

### 3.2. Analysis of the Association between NF-*κ*B and CYLD

**Figure 3:**
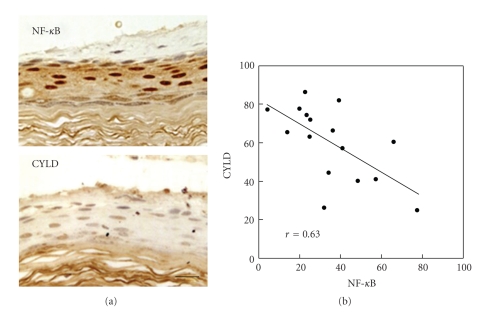
Analysis of the correlation between CYLD and activated NF-*κ*B in cholesteatoma. (a) The calculations were based on the immunohistochemical results of the expression rate of cells positive for CYLD and nuclear NF-*κ*B in the serial section. (b) The graph shows the correlation between CYLD and activated NF-*κ*B in cholesteatoma. The data indicated a significant inverse correlation between CYLD and activated NF-*κ*B expression (Pearson's correlation test; *r* = −0.630,* P* = .007).

To find the association between CYLD and NF-*κ*B, we examined the correlation between CYLD and activated NF-*κ*B expression. The calculations were based on the percentage of positively stained cells. Using Pearson's correlation test, we found a significant inverse correlation between CYLD and activated NF-*κ*B expression in cholesteatoma (Figure 3, *r* = −0.630, *P* = .007). 

### 3.3. Analysis of Clinical Aspects

Based on the overwhelming evidence indicating the roles of CYLD and activated NF-*κ*B in immune regulation, apoptosis, and bone resorption, we examined whether the expression levels of activated NF-*κ*B or CYLD are regulated according to clinical aspects, such as the presence of active otorrhea and state of bony destruction [6, 9]. The cholesteatoma groups in this study consisted of 6 cases with and 10 cases without active otorrhea at the time of treatment. We found no significant differences in expression levels of NF-*κ*B or CYLD between these two groups with and without active otorrhea (*P* = .309). 

 Based on operative findings, the above patients were classified retrospectively into two groups to determine the extent to which the cholesteatoma destroyed bone; the mild group had disruption of one ossicle, while the severe group had widespread bone destruction, including disruption of more than two ossicles and destruction of the sigmoid sinus, post wall of the external auditory canal, and the facial canal. We then compared the expression levels of CYLD and activated NF-*κ*B between the two groups. The degree of bone destruction was mild in 5 cases and severe in 11 cases in this study. No significant relationships were observed between the degree of bone destruction and the expression levels of CYLD (*P* = .769) or activated NF-*κ*B (*P* = .432).

## 4. Discussion

CYLD is a tumor suppressor gene mutated in familial cylindromatosis, also called “turban tumor syndrome,” an autosomal-dominant condition that confers a predisposition to multiple skin tumors [1]. The gene product, CYLD, is a DUB recently implicated in the negative regulation of the NF-*κ*B pathway, a transcription factor suggested to promote cell proliferation [1, 5]. The present study was performed to examine the possible role of CYLD expression in the pathogenesis of cholesteatoma. Specifically, we were interested in whether CYLD expression is altered in cholesteatoma and whether CYLD expression level affects the activity of NF-*κ*B in cholesteatoma. 

 The results of the present study indicated that the level of CYLD expression was significantly downregulated in cholesteatoma in comparison with normal RA skin. The primary function of DUBs is to remove single or multiple ubiquitin chains from different proteins/substrates. Recently, different specific substrates for CYLD have been identified, such as TRAF2/6, NEMO, and Bcl-3 from which CYLD could remove the ubiquitin chains by direct association [1, 10]. The removal of lysine63-(Lys63-) linked polyubiquitin chains from TRAF2 or TRAF6 by CYLD attenuates NF-*κ*B signaling, leading to programmed cell death [11]. Therefore, loss of the deubiquitinating activity of CYLD may be correlated with tumorigenesis. 

 Recently, reduced CYLD was also found in human carcinomas, such as hepatocellular carcinoma and colon cancer, as compared with nonneoplastic tissue [7]. Moreover, exposure of CYLD knockdown HeLa cells to anti-inflammatory drugs such as sodium salicylate or prostaglandin A1, which inhibit NF-*κ*B, abolished the protective knockdown of apoptosis, suggesting that loss of CYLD confers resistance to apoptosis through activation of NF-*κ*B [12]. CYLD is expressed in multiple tissues, but its precise biological function remains unclear. As noted above, a series of reports have shown that CYLD blocks signal transmission through the classical or alternative NF-*κ*B cascade by deubiquitinating the TNF receptor-associated factor 2 (TRAF2), TRAF6, and IKK-*γ* (NEMO) [1, 5, 6]. 

 However, a recent report showed that the loss of CYLD in keratinocytes of mice did not affect transcription induced by the classical p65–p50 NF-*κ*B dimers but had clear effects on Bcl-3-linked p50- or p52-dependent gene regulation [10]. Bcl-3 acts as a nuclear coactivator that switches the transcriptional properties of NF-*κ*B p50 and p52 homodimers from a repressive to an active state leading to cellular proliferation through activation of the cyclin D1 gene [10]. After translocation of Bcl-3 from the cytoplasm to the perinuclear region, CYLD removes K63-linked polyubiquitin chains from Bcl-3, and thus prevents translocation of Bcl-3 into the nucleus [10]. These data suggest that the mechanism of CYLD-mediated NF-*κ*B suppression may vary in different cell types. 

 In the present study, the cytoplasmic and perinuclear expression levels of CYLD suggest that CYLD may play a role in the deubiquitination of BCl-3 and/or TRAF in NF-*κ*B signaling within the cytoplasm or perinuclear region in keratinocytes of normal skin and cholesteatoma, in agreement with previously reported results. In addition, the present findings suggested that downregulation of CYLD expression, which is highly expressed in normal skin, may be involved in the pathogenesis of human cholesteatoma.

 The role of NF-*κ*B in epithelial keratinocytes has been described as being paradoxical in other cell types such as T cells and B cells [13]. For example, mice overexpressing Rel/p65 NF-*κ*B showed epidermal hypoplasia and growth inhibition [14], whereas a study on I*κ*B*α*-null mice showed epidermal keratinocyte hyperplasia and dermal infiltration of lymphocytes [15]. This variation in outcome of NF-*κ*B activation may reflect the cell types and differentiation state of the cells. Lizzul suggested that the antiproliferative effects of activated NF-*κ*B in normal epithelial cells without inflammation or apoptotic stimuli may be superior to its anti apoptotic effects [16].

 Activated NF-*κ*B, however, has an anti-apoptotic effect in proliferative skin disorders such as psoriasis. In normal murine skin, only basal keratinocytes can proliferate and differentiate into mature cells that undergo enucleation to generate the cornified layer. Therefore, NF-*κ*B is found in the cytoplasm (inactivated form) of only basal keratinocytes of normal skin [17]. Our immunohistochemical results of normal RA skin were in agreement with these findings. 

 In the present study, however, significant differences in NF-*κ*B expression pattern were observed between cholesteatoma and normal RA skin. In cholesteatoma, strong immunostaining for NF-*κ*B was seen mostly in the nucleus and was localized in not only the basal layer but also the suprabasal layers. This was in contrast to normal RA skin, which showed NF-*κ*B immunoreactivity mostly in the cytoplasm of cells in the basal epithelial layers. Some debate exists regarding the role of NF-*κ*B in cholesteatoma [2, 3]. The keratinocytes in cholesteatoma are clearly in an abnormal state, and one would anticipate changes in the epidermis and keratinocyte. Perhaps, this is why we observed in contrast control RA skin. That is, cholesteatoma may represent a chronic inflammatory state, and therefore, an imbalance exists between the anti-apoptotic role and cell cycle inhibitory role of NF-*κ*B, whereby the scale is tipped toward protection against cell death in the context of a constitutive cytokine-rich inflammatory milieu. This allows for the hyperproliferation seen in cholesteatoma. 

 Therefore, our results suggest that activated NF-*κ*B, which may provide protection against apoptosis, plays a role in the pathogenesis of cholesteatoma, unlike normal RA skin. Vasudevan reported that the tumor suppressor PTEN, which functions as a negative regulator of the phosphorylated Akt-mediated cell survival pathway in cholesteatoma, is downregulated by activated NF-*κ*B [18, 19]. These findings also support the results of the present study. To elucidate the role of CYLD in the expression of activated NF-*κ*B in human cholesteatoma, we examined the correlation between CYLD and activated NF-*κ*B expression in cholesteatoma. We found a significant inverse correlation between CYLD and NF-*κ*B in cholesteatoma, suggesting that NF-*κ*B activation is inhibited in cases with normal CYLD function but that it tends to be overexpressed in the absence of functional CYLD. In addition, our results support the possibility that NF-*κ*B activation accompanied by loss of CYLD may be an important step in the development and/or progression of cholesteatoma. We also examined the relationships between CYLD/NF-*κ*B and clinical data, such as the presence of active otorrhea and degree of bone destruction, but no such relationships were found in the present study. In addition to the critical role of CYLD in the NF-*κ*B pathway, CYLD has the ability to function as a negative regulator of the JNK signaling pathway, which plays an important role in cell survival in human cholesteatoma. The precise mechanisms underlying the downregulation of CYLD in cholesteatoma remain to be elucidated. Nevertheless, our data suggest the potential role of CYLD inactivation in cholesteatoma epithelium via multiple mechanisms, ranging from genetic alteration to epigenetic silencing.

## 5. Conclusions

We found that CYLD expression was lower and activated NF-*κ*B expression was higher in cholesteatoma epithelium in comparison to normal RA skin. These observations suggest that the activation of NF-*κ*B and downregulation of CYLD may be involved in the cellular hyperplasia in patients with cholesteatoma.
